# Mapping the landscape: A bibliometric perspective on autophagy in spinal cord injury

**DOI:** 10.1097/MD.0000000000038954

**Published:** 2024-07-19

**Authors:** Fei Wang, Songou Zhang, Yangjun Xu, Wei He, Xiang Wang, Zhongwei He, Jinxiang Shang, Zhang Zhenyu

**Affiliations:** a Department of Orthopedic Surgery, Shaoxing People’s Hospital, Zhejiang University, School of Medicine, Shaoxing, Zhejiang Province, China; b Ningbo University, School of Medicine, Ningbo, Zhejiang Province, China; c School of Medicine, Shaoxing University, Shaoxing City, Zhejiang Province, China; d Department of Orthopedic Surgery, Shaoxing People’s Hospital, Zhejiang University, School of Medicine, Shaoxing, Zhejiang Province, China; e Department of Thoracic Surgery, Shaoxing People’s Hospital, Shaoxing, Zhejiang Province, China; f Department of Orthopedic, Affiliated Hospital of Shaoxing University, Shaoxing, Zhejiang Province, China.

**Keywords:** autophagy, bibliometric, mTOR, spinal cord injury

## Abstract

**Background:**

Spinal cord injury (SCI) is a severe condition that often leads to persistent damage of nerve cells and motor dysfunction. Autophagy is an intracellular system that regulates the recycling and degradation of proteins and lipids, primarily through lysosomal-dependent organelle degradation. Numerous publications have highlighted the involvement of autophagy in the secondary injury of SCI. Therefore, gaining a comprehensive understanding of autophagy research is crucial for designing effective therapies for SCI.

**Methods:**

Dates were obtained from Web of Science, including articles and article reviews published from its inception to October 2023. VOSviewer, Citespace, and SCImago were used to visualized analysis. Bibliometric analysis was conducted using the Web of Science data, focusing on various categories such as publications, authors, journals, countries, organizations, and keywords. This analysis was aimed to summarize the knowledge map of autophagy and SCI.

**Results:**

From 2009 to 2023, the number of annual publications in this field exhibited wave-like growth, with the highest number of publications recorded in 2020 (44 publications). Our analysis identified Mei Xifan as the most prolific author, while Kanno H emerged as the most influential author based on co-citations. Neuroscience Letters was found to have published the largest number of papers in this field. China was the most productive country, contributing 232 publications, and Wenzhou Medical University was the most active organization, publishing 39 papers.

**Conclusion:**

We demonstrated a comprehensive overview of the relationship between autophagy and SCI utilizing bibliometric tools. This article could help to enhance the understanding of the field about autophagy and SCI, foster collaboration among researchers and organizations, and identify potential therapeutic targets for treatment.

## 1. Introduction

Spinal cord injury (SCI) is a debilitating condition that results in permanent motor dysfunction and sensory disturbance. Currently, approximately 20 million cases has been discovered globally. The incidence of SCI continues to rise in both developed and non-developed countries. This trend is expected to persist in the future.^[1]^ SCI leads to the loss of autonomic control over bowel, urinary bladder, and sexual function,^[2]^ significantly impacting patients’ daily lives. The pathological process of SCI involves both primary mechanical injury and secondary injury, with the latter being the primary cause of disability. Secondary injury encompasses oxidative stress reactions, such as the production of oxyradicals and lipid peroxidation aggregation,^[3]^ as well as spinal cord edema, vessel remodeling, ischemia, blood-spinal cord barrier damage,^[4]^ bleeding, neuroinflammation,^[5]^ ion disturbance,^[6]^ and glutamate excitatory toxicity.^[7]^ These factors contribute to damage to afferent neurons and motor nerves, resulting in sensory and motor disturbances. Current treatments for SCI include drug therapy, surgery, and rehabilitation, but none of these approaches can achieve a complete cure. It may be attributed to weak regeneration of spinal cord nerve^[8,9]^ and excessive apoptosis and autophagy due to the accumulation of pro-inflammatory factors.^[10]^ numerous studies have explored apoptosis. Thus, growing attention is now being paid to autophagy as a potential treatment avenue.

Autophagy is a cellular process where damaged organelles are degraded through lysosome-dependent pathways in eukaryotic cells under conditions of nutritional deficiency or oxidative stress. It plays a crucial role in maintaining internal environmental stability.^[11]^ Autophagy is also involved in various cellular activities, including quality control, early development, and cell differentiation.^[12]^ It has both beneficial and harmful effects: it enables the utilization of damaged organelles and proteins but excessive induction can lead to autophagic cell death, which impairs cells and differs from apoptosis-induced cell death.^[13]^ Autophagy is generally considered as a mechanism for promoting cell survival.^[14]^ However, autophagic cell death, which is non-apoptotic programmed cell death, occurs in dying cells and is mediated by autophagy rather than induced by it.^[13]^ Apoptotic cell death depends on the activation of caspases^[15]^ and clearance of cell debris by surrounding cells, while autophagy produces autophagosomes that are eliminated by lysosomes independently of caspase activation.^[16]^ Autophagic cell death can damage brain neurons in cases of cerebral trauma and cerebral infarction.^[17]^ Activation of the protein kinase B (AKT)/rapamycin (mTOR) pathway to attenuate autophagy has been shown to promote motor function recovery.^[18]^ However, relevant publications have demonstrated that autophagy protects nerve cells from death following SCI.^[19,20]^ Thus, proper regulation of autophagy is crucial for motor function recovery and nerve cell regeneration at different stages after SCI. Different treatments can be used to achieve the appropriate degree of autophagy based on the varying levels observed during different periods after SCI.

Currently, the death of nerve cells in SCI has been known to occur through multiple pathways, including necroptosis, apoptosis, pyroptosis, and autophagy. While autophagy has received less attention, it still holds promise as a target for SCI treatment. Necroptosis is a passive and uncontrolled form of cell death, apoptosis has been extensively studied without significant effective treatments, and our understanding of pyroptosis in SCI remains limited. With the important role of autophagy discovered in SCI, it represents a hopeful therapeutic target.

Bibliometric analysis is an effective and widely utilized tool for collecting vast amounts of information on publications within a specific field.^[21]^ This strategy allows for the visualization and analysis of selected papers, including summarizing the number of publications in a given field, assessing the publication landscape of relevant journals, identifying key organizations and authors, and aiding researchers in understanding the current hotspots and trends. Additionally, it broadens research perspectives, assists in identifying reputable journals, and facilitates collaboration opportunities. Furthermore, bibliometric analysis provides valuable insights into regulating autophagy for SCI treatment.

In this article, we have compiled critical information from screened publications on autophagy in SCI. It included annual publications, authors, co-cited authors, journals, countries, organizations, references, and keywords. We presented a network of related papers aiming to provide a clear map of autophagy in SCI. Our objective was to advance research progress in autophagy and SCI through this comprehensive analysis.

## 2. Methods

### 2.1. Date source and search strategy

Web of Science (WOS) is a comprehensive database that covers a wide range of publications from various fields,^[22]^ encompassing over 12,000 publications.^[23]^ With its extensive time coverage compared to other databases such as Scopus and PubMed, WOS is widely recognized as suitable for bibliometric analysis.^[24]^ We conducted a search in the Science Citation Index Expanded and Social Science Citation Index within the time span of 1985 to 2023. The search formula used was Topic = (“autophagy”) AND Topic = (“spinal cord injury”). We restricted the search to English-language papers and included only articles and review articles. We screened the articles based on titles, abstracts, and full texts, excluding any irrelevant papers. The publication dates were exported as plain text file” and record content contained “Full Record and Cited References.”

### 2.2. Date analysis

For bibliometric analysis, VOSviewer and Citespace software were employed using the exported data. Additionally, GraphPad Prism and SCImago were utilized to analyze the geographic distribution of countries and visualize the proportion of country-wise publications and organization-wise publications. VOSviewer, a Java-based software, aided in generating visualization maps with clusters, time, and density, enabling the construction of maps for authors, coauthors, journals, countries, organizations, and keywords. Citespace, another Java-based software created by Chaomei Chen,^[25]^ was used to generate lists of burst keywords and link strength over time.

### 2.3. Review method

**Figure 1.**
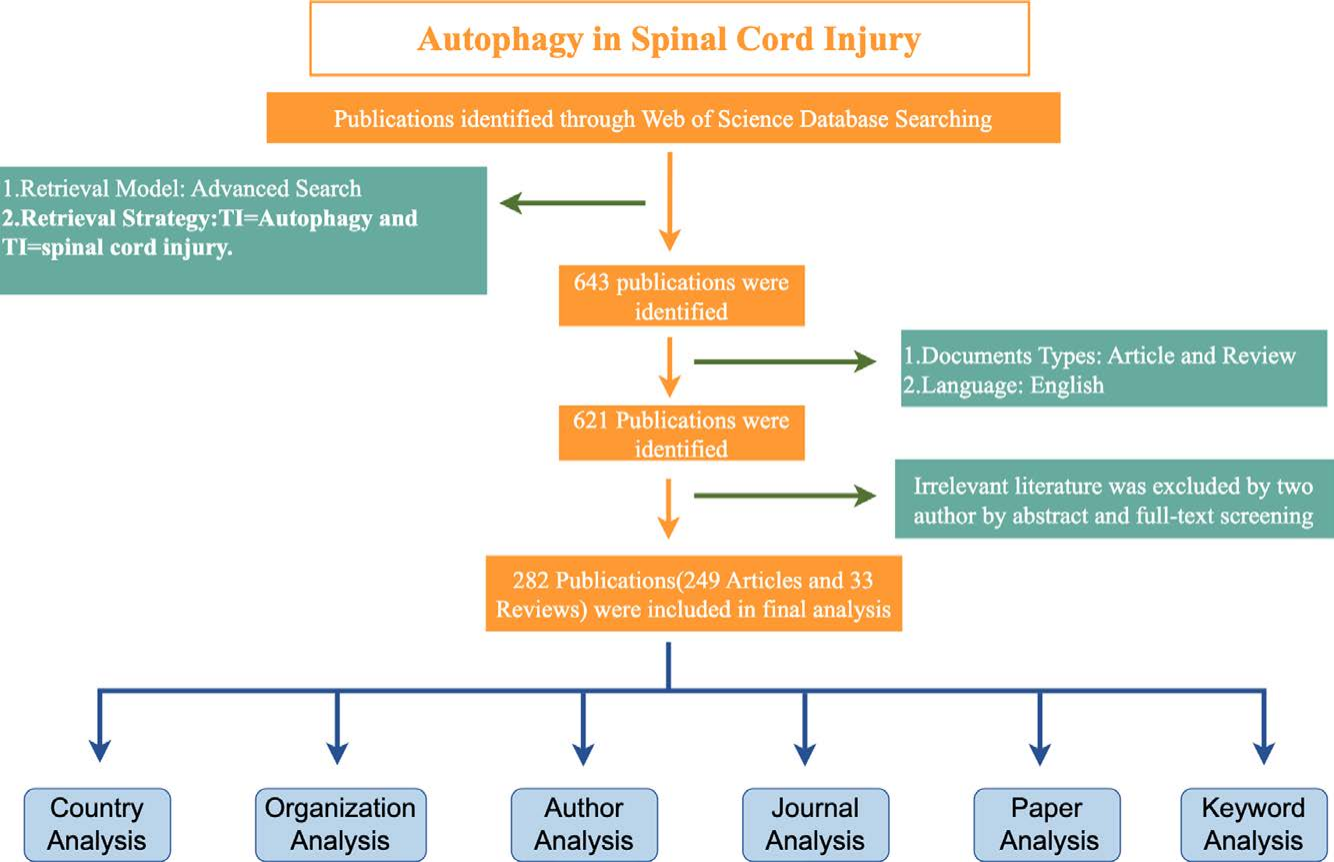
Publication screening flowchart.

The literature search was conducted in WOS, resulting in the selection of 643 publications. After excluding duplicates and irrelevant articles, we were left with 621 publications consisting solely of articles and review articles. Ultimately, 282 papers that were closely relevant to autophagy and SCI remained for bibliometric analysis. These 282 papers were chosen after careful elimination of duplications and publications not directly related to autophagy and SCI. The flowchart illustrating the process of literature screening is presented in Figure 1.

## 3. Results

### 3.1. Publication summary

**Figure 2.**
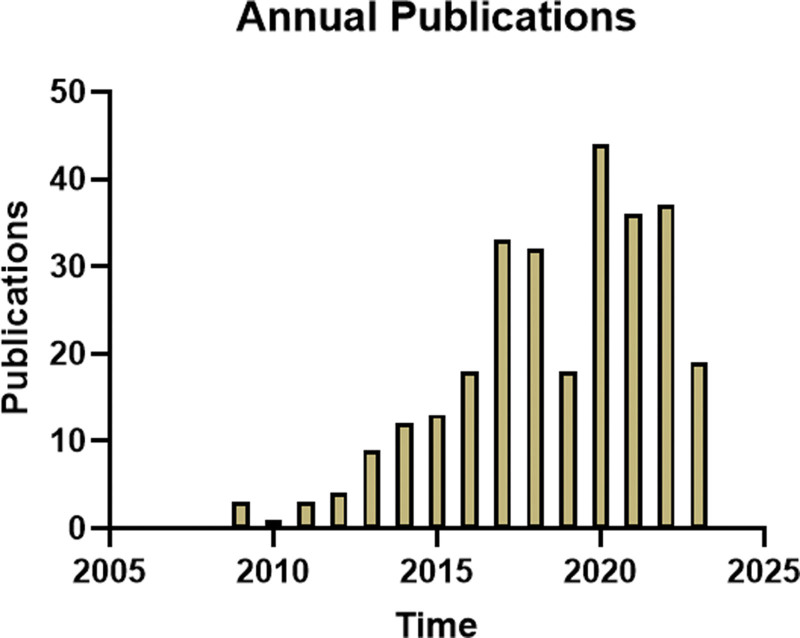
Annual output of autophagy in SCI. SCI = spinal cord injury.

A total of 282 publications relating to autophagy and SCI were identified from 2009 to 2023, comprising 249 articles and 33 reviews. The annual trend of publications is depicted in Figure 2, with the first article being published in 2009. The lowest number of publications occurred in 2010 (1 publication), while the highest number was recorded in 2020 (44 publications). In recent years, there has been a fluctuating increase in the annual number of publications.

### 3.2. Authors

**Figure 3.**
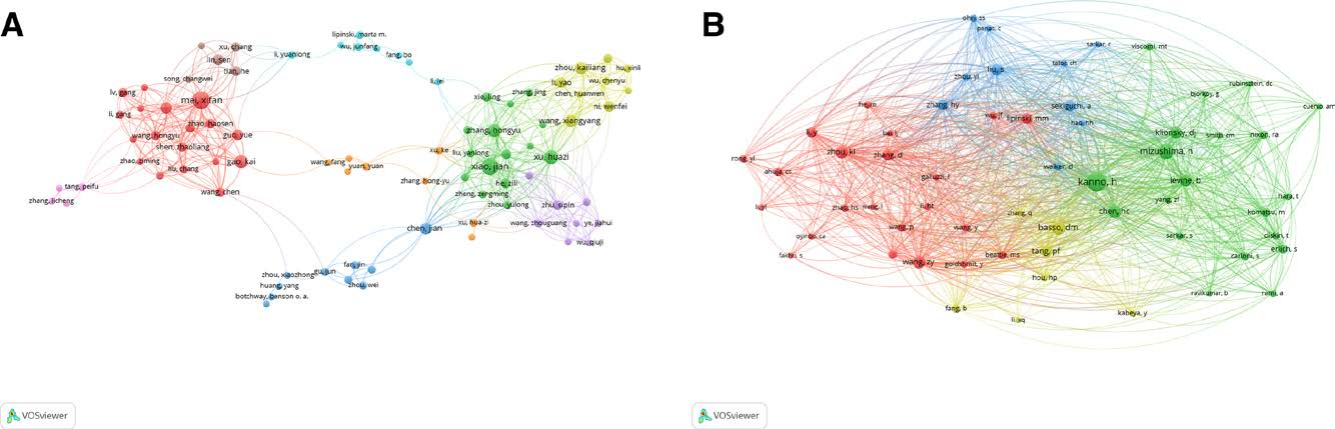
The visualization of authors (A) and co-cited authors (B) on research of autophagy in SCI. SCI = spinal cord injury.

A total of 1527 authors were mentioned across the 282 publications. Based on individual publication counts, the top 10 authors were selected, and their respective number of papers, institutions, locations, total citations, average citations, and total link strength are displayed in Table 1. Mei, Xifan is the most prolific author with 18 papers, followed by Xiao, Jian (17 papers), Xu, Huazi (12 papers), Gao, Kai (10 papers), and Zhang, Hongyu (10 papers). Additionally, 108 authors contributed 3 or more papers and were considered for author collaboration analysis (Fig. 3A).

**Table 1:** The top 10 authors in the field of autophagy and spinal cord injury.

Rank	Author	Count	Institution	Location	Total citations	Average citations	Total link strength
1	Mei, Xifan	18	Jinzhou Med Univ	China	573	31.83	23
2	Xiao, Jian	17	Wenzhou Med Univ	China	498	29.29	31
3	Xu, Huazi	12	Wenzhou Med Univ	China	412	34.33	24
4	Gao, Kai	10	Xi An Jiao Tong Univ，Jining 1 Peoples Hosp	China	392	39.20	9
5	Zhang, Hongyu	10	Wenzhou Med Univ	China	203	20.30	18
6	Wang, Xiangyang	9	Wenzhou Med Univ	China	156	17.33	21
7	Zhou, Kailiang	9	Wenzhou Med Univ	China	227	25.22	21
8	Chen, Jian	8	Three Gorges Cent Hosp Chongqing	China	195	24.37	8
9	Yuan, Yajiang	8	Jinzhou Med Univ	China	216	27.00	12
10	Kanno, Haruo	7	Tohoku Med & Pharmaceut Univ	Japan	557	79.57	7

### 3.3. Co-cited authors

A total of 8470 authors were co-cited in connection with the 282 articles, with 59 authors being co-cited at least 20 times (Fig. 3B). The most co-cited authors were Kanno, H (194 co-citations), Mizushima, N (123 co-citations), and Basso, DM (94 co-citations). Table 2 lists the top 10 co-cited authors, who accrued at least 65 citations.

**Table 2:** Top 10 co-cited author.

Rank	Co-cited author	Total citations	Institution	Location
1	Kanno, H	194	Tohoku Med & Pharmaceut Univ	Japan
2	Mizushima, N	123	Univ Tokyo, Grad Sch Med, Dept Biochem & Mol Biol	Japan
3	Basso, DM	94	Ohio State Univ	USA
4	Chen, HC	84	Jilin Univ	China
5	Wang, ZY	78	Shandong Agr Univ	China
6	Zhou, KL	78	Wenzhou Med Univ	China
7	Klionsky, DJ	77	Univ Michigan	USA
8	Levine, B	72	Univ Texas Southwestern Med Ctr Dallas	USA
9	Liu, S	67	Virginia Commonwealth Univ	USA
10	Tang, PF	67	Department of Orthopedic Medicine	China

### 3.4. Journals

**Figure 4.**
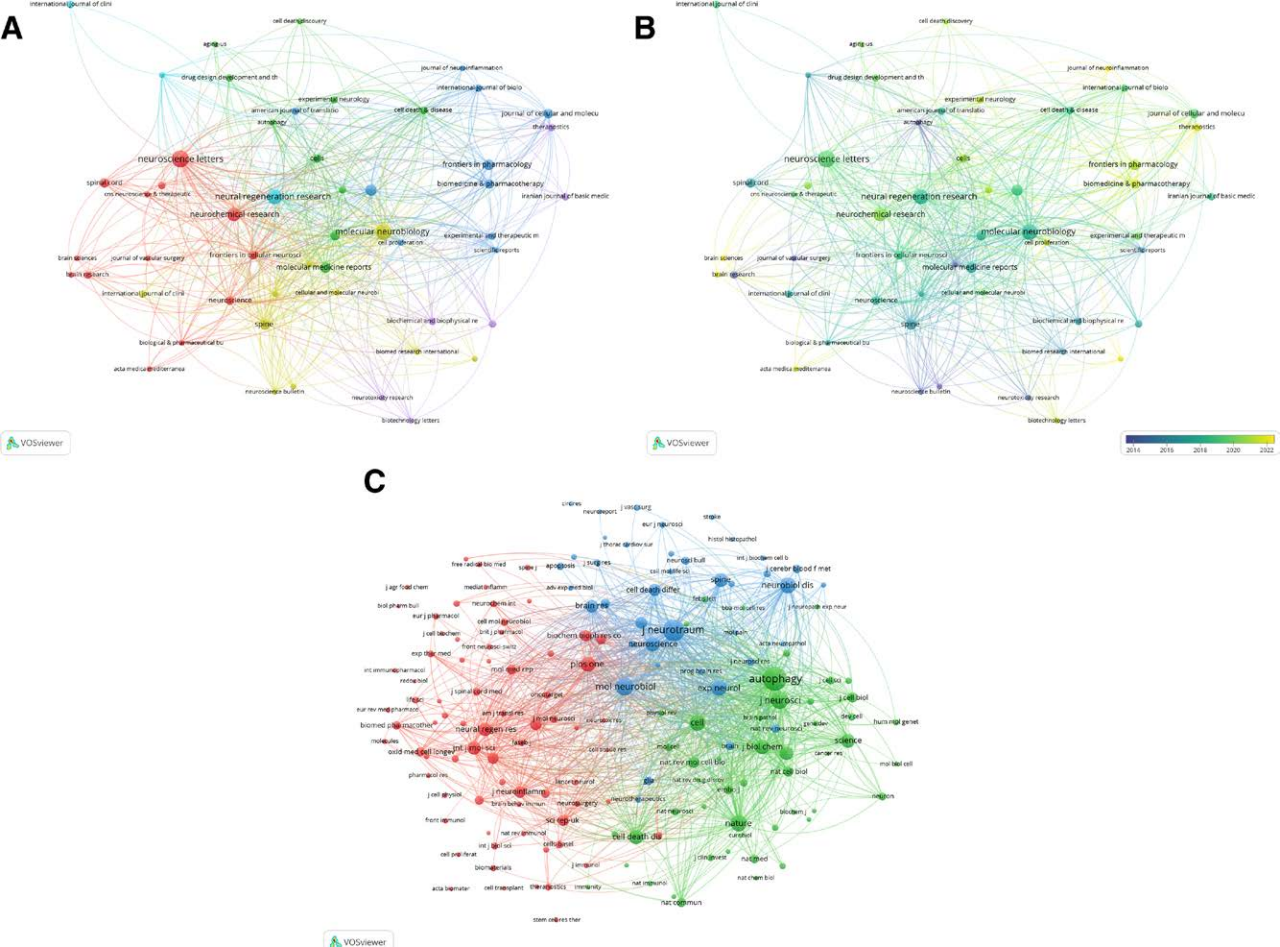
The visualization of journals (A, B) and co-cited journals (C) on research of autophagy in SCI. SCI = spinal cord injury.

The 282 articles were published in a total of 136 journals. Table 3 presents the top 12 journals based on publication count, including their respective number of publications, citations, impact factor (IF) for 2022, and Journal Citation Reports division for 2022. Among these top journals, 2 were classified as Q1 in the Journal Citation Reports, 7 as Q2, and 3 as Q3. Neuroscience Letters published the most relevant publications on autophagy and SCI, with a total of 16 articles. Moreover, Oxidative Medicine and Cellular Longevity had the highest IF among these top 12 journals. For journal relation analysis based on clusters and times, journals that published at least 2 papers were included (Fig. 4A and B).

**Table 3:** The top 12 productive journals correlated with autophagy and spinal cord injury.

Rank	Journal	Count	Percentage	Total citations	IF (2022)	JCR division (2022)
1	Neuroscience Letters	16	5.67%	244	2.5	Q3
2	Molecular Neurobiology	14	4.96%	690	5.1	Q2
3	Neural Regeneration Research	12	4.26%	231	6.1	Q2
4	Neurochemical Research	10	3.55%	191	4.4	Q2
5	Oxidative Medicine and Cellular Longevity	7	2.48%	162	7.3	Q2
6	Spine	6	2.13%	252	3.0	Q3
7	Molecular Medicine Reports	6	2.13%	122	3.4	Q3
8	Frontiers in Pharmacology	6	2.13%	77	5.6	Q1
9	Journal of Neurotrauma	5	1.77%	211	4.2	Q2
10	Journal of Cellular and Molecular Medicine	5	1.77%	193	5.3	Q2
11	Spinal Cord	5	1.77%	74	2.2	Q3
12	Biomedicine & Pharmacotherapy	5	1.77%	25	7.5	Q1

JCR = Journal Citation Reports.

### 3.5. Co-cited journals

A total of 1806 co-cited journals were associated with the 282 articles, with 164 journals being co-cited at least 20 times (Fig. 4C). Autophagy was the most co-cited journal (551 co-citations), followed by Journal of Neurotrauma (468 co-citations) and Molecular Neurobiology (318 co-citations). Of the top 10 co-cited journals, 6 were classified as Q1, 3 as Q2, and one as Q3. Additionally, 70% (7/10) of these top co-cited journals had an IF over 5.

### 3.6. Country

**Figure 5.**
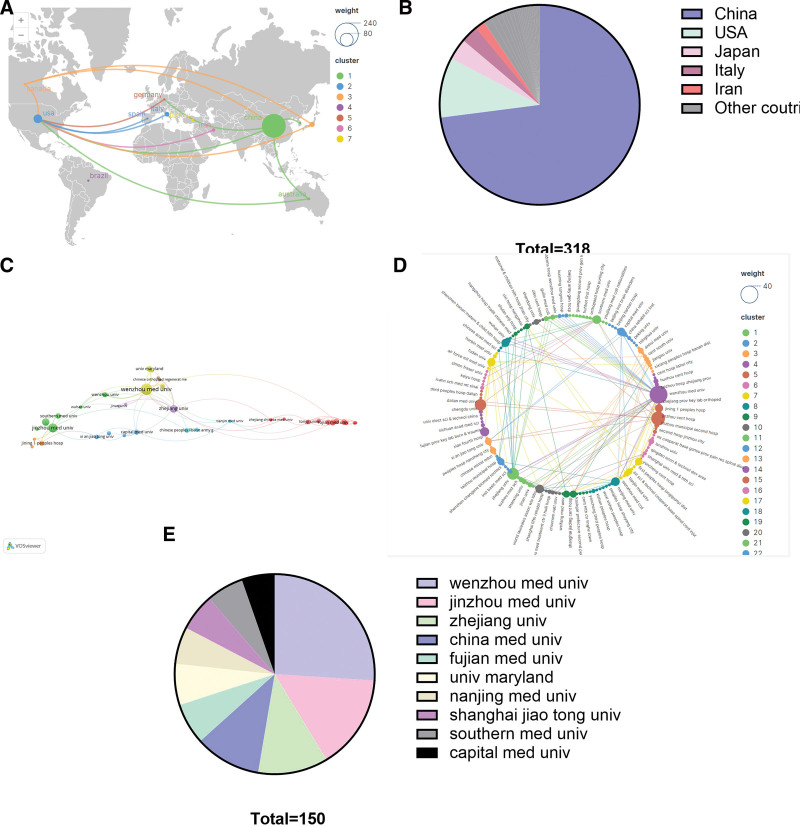
The visualization of country (A and B) and organization (C–E) on research of autophagy in SCI. SCI = spinal cord injury.

The 282 publications involved contributions from 24 countries. Table 4 presents the top 10 countries, with China leading in the number of publications (232 publications), followed by the USA (31 publications) and Japan (11 publications). Japan possessed the highest average citation count, followed by the USA and Italy. The international collaboration between countries, involving collaborations with at least 2 other countries, is illustrated in Figure 5A, while Figure 5B displays the proportion of each country’s publications.

**Table 4:** The most co-cited journals associated with autophagy and spinal cord injury.

Rank	Co-cited journal	Total citations	IF (2022)	JCR division (2022)
1	Autophagy	551	13.3	Q1
2	J Neurotraum	468	4.2	Q2
3	Mol Neurobiol	318	5.1	Q2
4	J Neurosci	300	5.3	Q1
5	Nature	269	64.8	Q1
6	Neurobiol Dis	255	6.1	Q1
7	Cell	244	64.5	Q1
8	Exp Neurol	240	5.3	Q1
9	PLOS One	227	3.7	Q2
10	Neuroscience	212	3.3	Q3

JCR = Journal Citation Reports.

### 3.7. Organization

A total of 318 organizations were associated with the 282 publications. Table 5 lists the top 10 productive organizations, all of which are high schools, with 90% (9/10) located in China. Wenzhou Medical University ranked first in productivity with 39 publications, followed by Jinzhou Medical University with 23 publications and Zhejiang University with 17 publications. Figure 5C shows organizational collaborations based on 38 organizations collaborating at least 3 times, Figure 5E illustrates the proportion of the top 10 organizations, and Figure 5D displays a radial analysis of the collaborations between all organizations related to the 282 papers.

**Table 5:** The top 10 productive countries in the field of autophagy and spinal cord injury.

Rank	Country	Documents	Percentage	Total citations	Average citations
1	China	232	82.27%	5159	22.24
2	USA	31	10.99%	1703	54.94
3	Japan	11	3.90%	694	63.09
4	Italy	9	3.19%	316	35.11
5	Iran	5	1.77%	77	15.40
6	Australia	4	1.42%	129	32.25
7	South Korea	3	1.06%	97	32.33
8	Turkey	3	1.06%	46	15.33
9	Brazil	2	0.71%	19	9.50
10	Canada	2	0.71%	63	31.50

### 3.8. Papers

Among the 282 publications, 38 publications received over 50 citations. Table 6 summarizes the top 10 cited publications, with the most cited paper being “Health benefits of anthocyanins and molecular mechanisms: Update from the recent decade” by Li, Daotong (273 citations). Furthermore, 14 papers reached 100 citations.

**Table 6:** The top 10 productive organizations published literature related to autophagy and spinal cord injury.

Rank	Organization	Country	Documents	Total citations	Average citations
1	Wenzhou Med Univ	China	39	1022	26.21
2	Jinzhou Med Univ	China	23	604	26.26
3	Zhejiang Univ	China	17	456	26.82
4	China Med Univ	China	16	320	20.00
5	Fujian Med Univ	China	10	208	20.80
6	Univ Maryland	USA	10	886	88.60
7	Nanjing Med Univ	China	9	250	27.78
8	Shanghai Jiao Tong Univ	China	9	160	17.78
9	Southern Med Univ	China	9	164	18.22
10	Capital Med Univ	China	8	88	11.00

### 3.9. Co-cited reference

**Figure 6.**
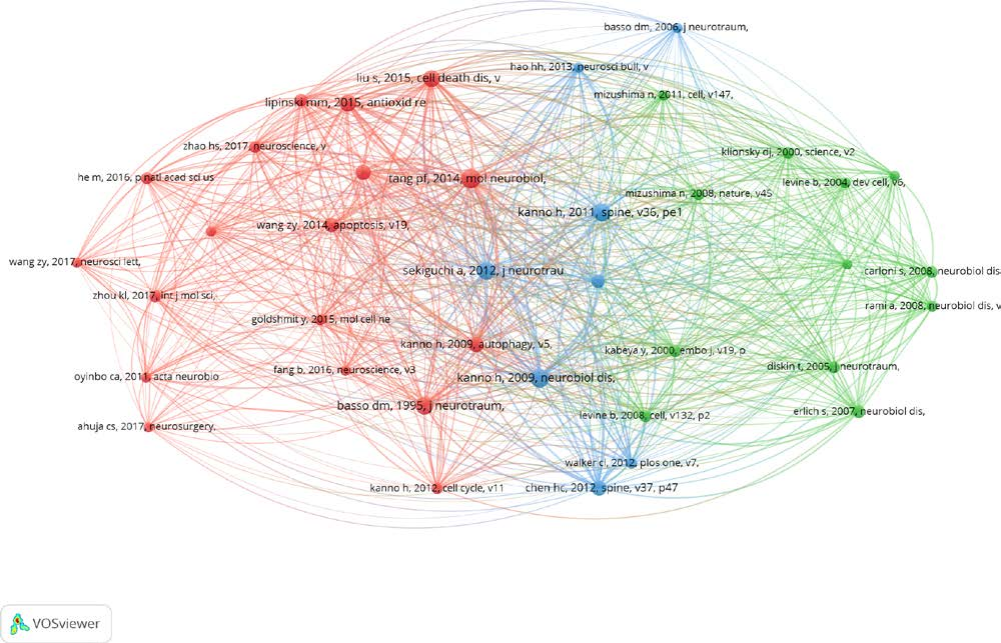
The visualization of co-cited reference.

A total of 10,837 co-cited references were related to the 282 papers. Figure 6 includes 38 publications with at least 20 co-citations. Table 7 showcases the top 10 co-cited papers, with the paper by Tang, Peifu in 2014 receiving the highest number of co-citations (66 co-citations), followed by Kanno, Haruoaruo in 2009 (63 co-citations) and Kanno, Haruo in 2011 (59 co-citations).

**Table 7:** Top 10 most citied paper.

Rank	Paper	Citations	Journal	Author	Institution	Location
1	Health benefits of anthocyanins and molecular mechanisms: Update from recent decade	273	Critical Reviews in Food Science and Nutrition	Li, Daotong	China Agr Univ	China
2	Systemic bisperoxovanadium activates Akt/mTOR, reduces autophagy, and enhances recovery following cervical spinal cord injury	176	PLOS One	Walker, Chandler L	Indiana Univ Sch Med	USA
3	Disrupted autophagy after spinal cord injury is associated with ER stress and neuronal cell death	159	Cell Death & Disease	Liu, S	Univ Maryland	USA
4	Rapamycin promotes autophagy and reduces neural tissue damage and locomotor impairment after spinal cord injury in mice	153	Journal of Neurotrauma	Sekiguchi, Akira	Tohoku Univ	Japan
5	Resveratrol protects against spinal cord injury by activating autophagy and inhibiting apoptosis mediated by the SIRT1/AMPK signaling pathway	141	Neuroscience	Zhao, Haosen	Jinzhou Med Univ	China
6	Autophagy reduces neuronal damage and promotes locomotor recovery via inhibition of apoptosis after spinal cord injury in rats	139	Molecular Neurobiology	Tang, Peifu	Chinese Peoples Liberat Army Gen Hosp	China
7	Reactive astrocytes undergo M1 microglia/macrophages-induced necroptosis in spinal cord injury	139	Molecular Neurodegeneration	Fan, Hong	Fourth Mil Med Univ	China
8	Function and mechanisms of autophagy in brain and spinal cord trauma	136	Antioxidants & Redox Signaling	Lipinski, Marta M.	Univ Maryland, Sch Med	USA
9	Regulation of autophagy and ubiquitinated protein accumulation by bFGF promotes functional recovery and neural protection in a rat model of spinal cord injury	127	Molecular Neurobiology	Zhang, Hong-Yu	Wenzhou Med Coll	China
10	Autophagy induction stabilizes microtubules and promotes axon regeneration after spinal cord injury	124	Proceedings of the National Academy of Sciences of the United States of America	He, Miao	Chinese Acad Sci	China

FGF = fibroblast growth factor.

### 3.10. Keywords

**Figure 7.**
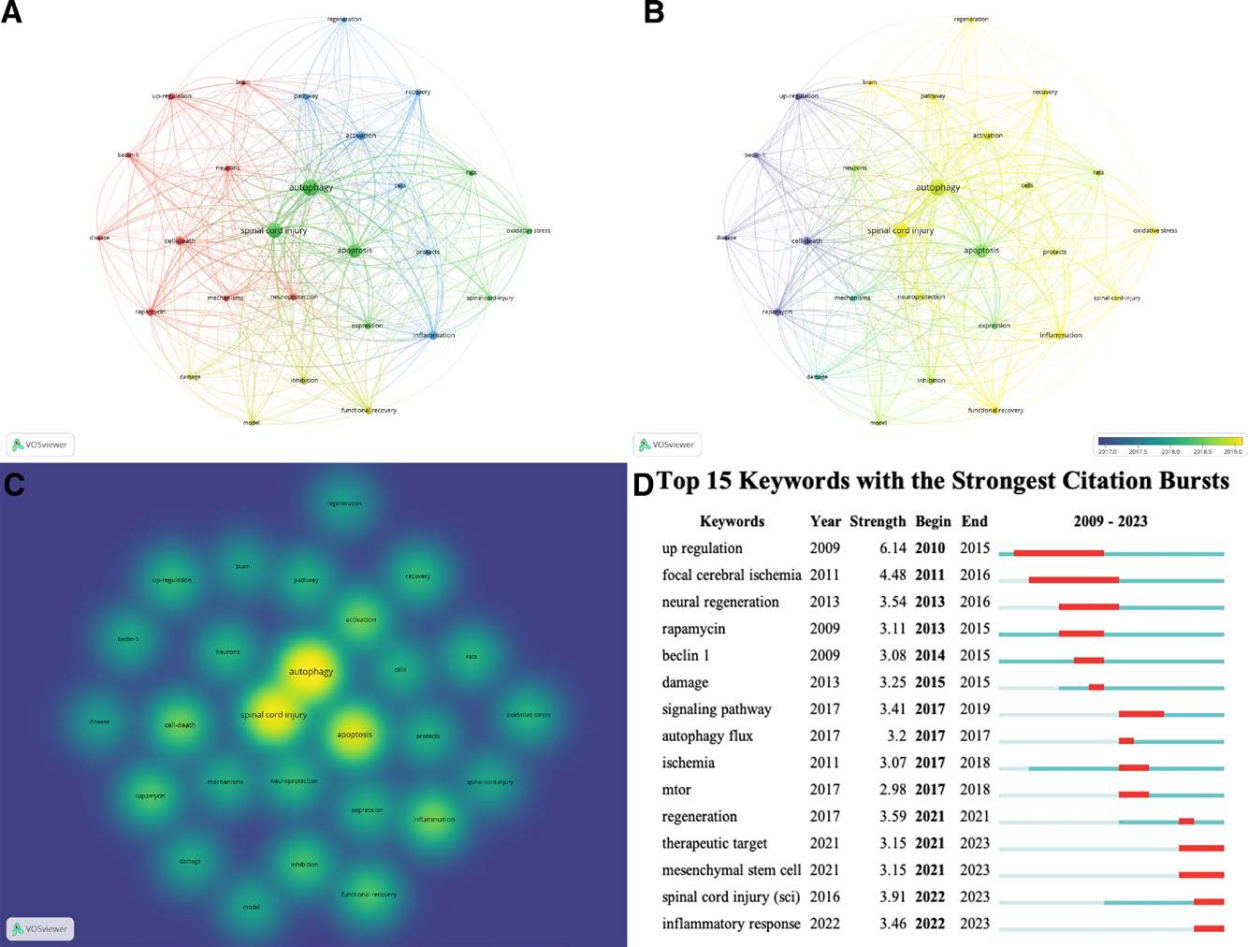
The visualization of keywords (A, B, C) and keywords burst (D).

A total of 1170 keywords were identified through co-occurrence analysis, with 59 keywords appearing at least 10 times and 27 keywords appearing at least 20 times. The most frequently occurring keyword was autophagy (n = 204), followed by SCI (n = 165), apoptosis (n = 130), inflammation (n = 62), and cell death (n = 60). The visual analysis, based on the 27 keywords appearing at least 20 times, is presented in Figure 7. These keywords were divided into 4 clusters (Fig. 7A). The different clusters represent diverse categories: Cluster 1 (red) focused on beclin-1, cell death, disease, mechanisms, neurons, neuroprotection, rapamycin, and up-regulation; Cluster 2 (green) encompassed apoptosis, autophagy, expression, oxidative stress, rats, SCI, and spinal-cord-injury; Cluster 3 (blue) included activation, cells, inflammation, pathway, protects, recovery, and regeneration; Cluster 4 (yellow) consisted of damage, functional recovery, inhibition, and model. Figure 7B and C depicts correlation analyses based on time and density. Burst keywords display the frequency of keywords in specific time periods, providing information about research hotspots from the past to the present and indicating current focal points as well as potential future breakthroughs. The most relevant burst keywords are listed in Figure 7D.

## 4. Discussion

From a bibliometric perspective, this study provided a systematic analysis of autophagy and SCI, yielding visual and comprehensive results. These findings not only reflected the overall research trends in the field but also uncovered current hotspots and cutting-edge developments.

### 4.1. General information

Regarding annual publications, the first publication in this field was published in 2009, and there has been a fluctuating increase in annual publications since then. The highest number of publications occurred in 2020, indicating a growing interest in the relationship between autophagy and SCI. Over the past 14 years, there have been 5 years with more than 30 publications. As the understanding of autophagy mechanisms, such as mTOR^[26]^ and microRNA,^[27]^ deepens, we can expect further research in this area to be published.

Among the most productive authors in this field, Mei Xifan, Xiao Jian, Xu Huazi, Gao Kai, and Zhang Hongyu have each contributed at least 10 papers. Notably, Xiao Jian, Xu Huazi, and Gao Kai are affiliated with Wenzhou Medical University, suggesting that scholars from this institution are particularly interested in autophagy in SCI. Mei Xifan, from Jinzhou Medical University, has published the highest number of papers (18) and focuses on the regulation of the silent information regulator sirtuin 1/AMP-activated protein kinase (AMPK) signaling pathway and autophagy. Kanno H (194 co-citations) and Mizushima N (123 co-citations) are the most frequently co-cited authors in this field, both hailing from Japan, indicating the high quality of Japanese scholars’ contributions. It is worth noting that Kanno H ranks among the top authors. Additionally, top 2, top 3, and top 9 co-cited paper all were published by him, demonstrating his significant achievements and authority in the field of autophagy and SCI (Table 8). Additionally, Akira Sekiguchi (57 co-citations, rank 5) and Kanno Haruo, both affiliated with Tohoku University School of Medicine, have collaborated multiple times on autophagy and SCI research. In China, Tang Peifu, working at the Chinese People’s Liberation Army General Hospital, has made substantial contributions to autophagy and SCI research. Tang Peifu ranks among the top ten coauthors, has the sixth most cited papers, and is involved in the most co-cited papers, focusing on hydrogel, fracture, apoptosis, SCI, and autophagy. Similarly, Zhang Hongyu, employed at Wenzhou Medical University, has made significant contributions to this field, ranking fifth among authors, ninth among cited papers, and tenth among co-cited papers. Zhang Hongyu research primarily focuses on autophagy, SCI, and endoplasmic reticulum stress.

**Table 8:** Top 10 most co-cited papers.

Rank	Title	Author	Citation	Institution	Location
1	Autophagy reduces neuronal damage and promotes locomotor recovery via inhibition of apoptosis after spinal cord injury in rats	Tang, Peifu	66	Chinese Peoples Liberat Army Gen Hosp	China
2	Spinal cord injury induces upregulation of Beclin 1 and promotes autophagic cell death	Kanno, Haruo	63	Tohoku University School of Medicine	Japan
3	Induction of autophagy and autophagic cell death in damaged neural tissue after acute spinal cord injury in mice	Kanno, Haruo	59	Tohoku University School of Medicine	Japan
4	A sensitive and reliable locomotor rating scale for open field testing in rats	D M Basso	57	Department of Cell Biology	USA
5	Rapamycin promotes autophagy and reduces neural tissue damage and locomotor impairment after spinal cord injury in mice	Akira Sekiguchi	57	Tohoku University School of Medicine	Japan
6	Function and mechanisms of autophagy in brain and spinal cord trauma	Marta M Lipinski	50	University of Maryland School of Medicine	USA
7	Disrupted autophagy after spinal cord injury is associated with ER stress and neuronal cell death	S Liu	50	University of Maryland School of Medicine	USA
8	Autophagy is activated in injured neurons and inhibited by methylprednisolone after experimental spinal cord injury	Hsien-Chih Chen	44	Chang Gung Memorial Hospital	China
9	The role of autophagy in spinal cord injury	Haruo Kanno	43	Tohoku University School of Medicine	Japan
10	Regulation of autophagy and ubiquitinated protein accumulation by bFGF promotes functional recovery and neural protection in a rat model of spinal cord injury	Hong-Yu Zhang	41	Wenzhou Medical College	China

FGF = fibroblast growth factor.

Neuroscience Letters has published the highest number of relevant articles, while Molecular Neurobiology has the second highest number of publications and the highest total citation count, indicating its prominence in the field. Oxidative Medicine and Cellular Longevity had the highest IF(7.3) in 2022, suggesting its relative authority and reliability. Journal of Neurotrauma emerged as the second most co-cited journal and ninth in terms of publication volume, emphasizing its focus on nervous system trauma. Autophagy, a highly co-cited journal, explores autophagy mechanisms in various diseases. Furthermore, one of the earliest papers in this field was published in Autophagy in 2009.^[28]^ Nature and Cell, as renowned journals, have published exceptionally creative and impactful papers, making them frequently cited by scholars to support their theories and studies. In terms of country representation, Chinese researchers have published the majority of papers (82.27%), with China also receiving the highest total citations. However, the average number of citations per paper from China is the lowest among the top 3 productive countries (China, USA, and Japan), indicating that the quality of papers from China does not always reach the highest international standards. Conversely, Japan has the highest average number of citations, followed by the USA, suggesting the high quality of their publications related to autophagy and SCI.

With half of the most productive authors affiliated with Wenzhou Medical University, this institution has published the most papers on autophagy in SCI research. Although Chinese organizations account for a high proportion of productivity, the University of Maryland, the only American institution in the top 10, received nearly 3 times the average number of citations compared to Nanjing Medical University (the highest-cited Chinese organization).

The most cited paper in this field was “Health benefits of anthocyanins and molecular mechanisms: Update from the recent decade,”^[29]^ which offered insights into the molecular mechanisms of autophagy. Therefore, researchers preferred to cite this paper to explain the role of autophagy in SCI. Additionally, studies have shown that anthocyanins have neuroprotective effects^[30]^ and multifaceted effects,^[31]^ contributing to motor functional recovery in SCI. The most co-cited paper, “Autophagy Reduces Neuronal Damage and Promotes Locomotor Recovery via Inhibition of Apoptosis After SCI in Rats,” highlights the importance of inducing autophagy early to protect nerve cells and promote motor recovery through the inhibition of apoptosis.^[32]^

### 4.2. The hotspots and topical issues

Keywords are often considered condensed versions of full articles, and frequently appearing keywords are thought to represent hotspots in specific fields. Burst keywords connect keywords with time to reveal relevant hotspots in specific years. Based on the keywords and burst keywords, this section will discuss mesenchymal stem cells (MSCs), inflammatory response, and therapeutic targets.

#### 4.2.1. MSCs

MSCs, adherent fibroblast-like cells, were first discovered in human bone marrow in the 1960s.^[33]^ MSCs also discovered in various human tissues, including the liver,^[34]^ lungs,^[35]^ peripheral blood,^[36]^ periosteum,^[37]^ salivary glands,^[38]^ adipose tissue,^[39]^ synovial membrane,^[40]^ umbilical cord blood,^[41]^ blood vessel walls,^[42]^ Wharton jelly,^[43]^ dental pulp,^[44]^ placental tissue,^[45]^ tendon,^[46]^ skeletal muscle,^[47]^ menstrual blood,^[48]^ and amniotic fluid.^[49]^ What sets them apart from other stem cells is their ability to regulate immunity.^[50]^ The mechanisms behind the immunomodulation of MSCs include migrating to sites of inflammation or injury, changing the type of CD4+ T helper cell, inducing tolerogenic dendritic cells, regulating macrophage polarization and promoting their repair function, producing immunosuppressive soluble factors, and inducing immune tolerance.^[51]^ The inflammatory microenvironment plays a vital role in the immunoregulatory effects of MSCs and induces autophagy.^[52,53]^ Inflammatory factors such as immune interferon-γ and tumor necrosis factor-α primarily contribute to the autophagy of MSCs during inflammation.^[52]^ MSCs can inhibit inflammation through exosomes,^[54]^ promote axon regeneration and neuron survival by secreting brain-derived neurotrophic factor and β-nerve growth factor,^[55,56]^ and repair blood vessels by releasing angiogenic factors such as vascular endothelial growth factor, platelet-derived growth factor, and fibroblast growth factor.^[57]^ Inhibition of autophagy by knocking down Beclin 1 can significantly enhance the therapeutic effects on experimental autoimmune encephalomyelitis.^[58]^ Exosomes secreted by MSCs transport miRNA and immunosuppressive proteins to targeted cells, providing a treatment option without aging problems and rejection reactions.^[59,60]^ MSCs promote fusion of autophagosomes with lysosomes, and through excretion of exosomes, they can regulate autophagy via the AMPK/mTOR and AKT/mTOR pathways after SCI.^[61,62]^ The multiple functions of exosomes, including accelerating axonal regeneration and angiogenesis, modulating the inflammatory microenvironment and immune response, suppressing apoptosis, and maintaining the integrity of the blood-spinal cord barrier,^[63,64]^ have been revealed. Clinically, MSCs have been used to treat liver diseases,^[65]^ diabetes mellitus,^[66]^ and peripheral nerve injuries.^[67]^ Relevant research has discovered that TGF-β helps MSCs protect nerve regeneration.^[68]^ With more special functions of MSCs being discovered, the effectiveness of MSCs in treating SCI has been proven, and the question of how to improve their efficacy further remains to be addressed.

#### 4.2.2. Inflammatory response

Inflammation, a defensive response, aims to eliminate pathogens, damaged tissues, and cancer.^[69]^ However, excessive inflammation can damage tissue, and necrosis can perpetuate inflammation, leading to its persistence.^[70]^ Additionally, the inflammatory response is critical to the secondary injury of SCI.^[71]^ When a lesion occurs after SCI, immune cells pass through the blood-spinal cord barrier and migrate to the injury site to eliminate damaged tissue through phagocytosis and the release of anti-inflammatory factors. The polarization of macrophages and microglia plays an important role in the mechanism of SCI. Pro-inflammatory M1 macrophages appear early to phagocytose damaged tissue and promote the recruitment of leukocytes from peripheral blood. Conversely, anti-inflammatory M2 macrophages repair damaged tissues in the later stage.^[72,73]^ Therefore, fine-tuned alteration of macrophage polarization can alleviate inflammation and promote tissue repair.^[74]^

Recent research has shown that autophagy in macrophages can limit the development of inflammation, and the regulation of Beclin 1 can control the production of inflammatory factors.^[75]^ Mitophagy, a physiological metabolic process, eliminates damaged or dysfunctional mitochondria. Dysfunctional mitochondria can exacerbate inflammation, and proper mitophagy can balance inflammation through innate immunity responses, regardless of endogenous or exogenous sources of inflammation.^[76]^ In addition, reactive oxygen species (ROS) produced by mitochondria and damage-associated molecular patterns (DAMPs) can induce inflammatory responses by promoting inflammasome formation. Mitophagy can prevent excessive inflammatory responses induced by ROS and DAMPs.^[77]^ Hence, enhancing mitophagy can alleviate inflammation caused by ROS and DAMPs. Targeting mitophagy is also a promising direction for treating SCI.

AMPK is an important regulator of cellular energy and metabolism. Activation of the AMPK pathway induces autophagy, restricts inflammation metabolism,^[78]^ and attenuates pain induced by inflammation by inhibiting NF-κB activation and IL-1β expression.^[79]^ Selective induction of AMPK may be a reliable target for inflammation after SCI.

Inflammation after SCI is closely linked to autophagy. Liu discovered that Tripartite motif-containing 14 upregulates IL-12 and IL-23 expression to promote inflammation through crosstalk with lysine-specific demethylase 4D, inhibiting autophagic degradation epigenetically.^[80]^ Conversely, vascular endothelial growth factor,^[81]^ metformin,^[82]^ rapamycin,^[83]^ and curcumin^[84]^ activate autophagy to suppress inflammation. Therefore, identifying proper targets for autophagy could regulate the extent of inflammation, minimizing damage after SCI. The timing of inflammation after SCI presents a practical opportunity to modulate autophagy to alleviate inflammatory damage.

#### 4.2.3. Therapeutic targets

Autophagy plays a significant role in secondary injury and tissue repair, and targeted medicine can be more effective with fewer side effects. Therefore, it is necessary to identify proper targets for the treatment of SCI.

Rapamycin, an antifungal metabolite, has been found to inhibit immunity and proliferation in mammalian cells.^[85]^ It promotes the expression of microtubule-associated protein 1 light chain 3 and Beclin1 at the site of injury, inducing autophagy and increasing the number of neurons and astrocytes with light chain 3 in the spinal cord.^[32]^ The mammalian target of mTOR signaling pathway, an evolutionarily conserved serine/threonine kinase,^[86]^ regulates processes such as death, survival, metabolism, proliferation, and growth.^[87]^ This pathway, located in downstream effector of the phosphatidylinositol-3 kinase/AKT pathway, consists of 2 parts: mechanistic target of rapamycin complex 1 and mTORC2. The mechanistic target of rapamycin complex 1 pathway, which is sensitive to rapamycin, includes mTOR, regulatory-associated protein of mTOR, mammalian lethal with SEC13 protein 8, 40-kDa proline-rich Akt substrate, and DEP domain-containing mTOR-interacting protein.^[88]^ It controls the process of autophagy through various intracellular and extracellular cues, including stress, energy status, oxygen, amino acids, and growth factors.^[89]^ On the other hand, mTORC2, which is insensitive to rapamycin, is composed of mTOR, rapamycin-insensitive companion of mammalian target of rapamycin, mammalian lethal with SEC13 protein 8, DEP domain-containing mTOR-interacting protein, mammalian stress-activated protein kinase-interacting protein, and Protor1/2.^[90,91]^ It regulates cell survival, proliferation, and cytoskeleton remodeling.^[92]^ Autophagy is activated by AMPK, which senses cellular energy levels and balances cell metabolism. However, mTOR inhibits autophagy. Under conditions of glucose deprivation, AMPK activates autophagy by phosphorylating Ulk1.^[93]^ Phosphorylated Ulk1 is a key regulator of autophagy. Both AMPK and mTOR can regulate the phosphorylation of Ulk1 to control autophagy. Regulating autophagy has the potential to improve motor function, as dysregulation of autophagy is one of the causes of severe outcomes in SCI. Therefore, inhibiting or activating autophagy signaling pathways such as mTOR and AMPK can enhance functional recovery. Several studies have provided evidence for this theory, such as the discovery of the regulation of the AKT/mTOR/signal transducer and activator of transcription 3 pathway by adenosine triphosphate^[94]^ and the role of the transient receptor potential melastatin 7 channel in reducing blood-spinal cord barrier damage through the mTOR/Jumonji domain-containing protein-3 axis after SCI.^[95]^ In clinical practice, rapamycin analogues like deforolimus, everolimus, and temsirolimus are used to treat various cancers.^[96]^ With further research, more treatments for SCI will likely be discovered in the future.

Beclin 1, the first mammalian autophagy-related gene, was discovered by the Levine group in 1999. It is involved in multiple biological processes, including development, lifespan, and diseases. Beclin 1 is the mammalian homologue of yeast Atg6/vps30,^[97]^ although the amino acid sequences of Beclin 1 and Atg6/vps30 differ substantially (39.1% sequence similarity and 24.4% sequence identity). The Beclin 1 complex, located downstream of the mTOR signaling pathway, initiates the formation of autophagosomes and is regulated by the mTOR pathway to control autophagy.^[98]^ The tumor suppressing function of Beclin 1 has been well established.^[99]^ During autophagy, Beclin 1 is phosphorylated by Ulk1 and serves as a scaffold for the phosphatidylinositol-3 kinase complex, promoting the localization of autophagy-related proteins to the autophagic vacuole.^[100]^ Bcl-2, an antiapoptotic gene, interacts with Beclin 1 to regulate the balance between autophagy and apoptosis by inhibiting autophagy.^[101]^ The Beclin 1-Bcl-2 complex maintains this balance. Autophagy, induced by Beclin 1, is inhibited by the expression of Bcl-2. Nutrient starvation, a potent physiological inducer of autophagy, promotes the dissociation between Beclin 1 and its repressors.^[102]^ Moreover, cell death is weakly associated with autophagy when only Bcl-2 is expressed, while it is strongly linked to autophagy when Beclin 1 is activated alone. Many viruses have evolved mechanisms to inhibit autophagy, such as binding to Beclin-1, which inhibits autophagosome formation and impairs autophagy.^[103]^ Understanding how viruses inhibit Beclin 1 could provide insights into regulating autophagy in SCI treatment. Up-regulation of Beclin 1 and promotion of autophagic cell death have been observed after SCI. The expression of Beclin 1 is initially up-regulated at 4 hours, peaks at 3 days, and remains at a high level at 21 days.^[104]^ Therefore, inhibitors of autophagy may be potential and practical targets for treating SCI. Furthermore, Beclin 1 also mediates autophagy against apoptosis following SCI, playing an important role in the neuroprotection of the spinal cord.^[105]^

Axon regeneration after SCI is challenging due to the presence of extracellular inhibitory factors, limited axon growth capacity, and a lack of neurotrophic factors.^[106]^ Promoting axon regeneration requires activating growth programs and alleviating growth inhibitory pathways.^[10]^ In vitro studies have shown that inducing autophagy can reduce scar formation and promote axon regeneration at the lesion site after SCI by degrading the superior cervical ganglia protein 10, thereby stabilizing microtubules.^[107, 108]^ The cytoskeleton, composed of microtubules and actin, plays a crucial role in axon regeneration. Research has indicated that dysregulated cytoskeleton dynamics can result in a growth-incompetent structure, but pharmacological modulation can reverse this effect.^[109]^ The mechanism of axon regeneration is complex, and potential treatment targets include phosphatase and tensin homolog/mTOR,^[110]^ the mitogen-activated protein kinase pathway,^[111]^ and signal transducer and activator of transcription 3.^[112]^ Recently, researchers discovered that ROS-Scavenging Hydrogels, a new material designed to remove ROS could promote nerve regeneration and improve the microenvironment to restore motor function after SCI.^[113]^ As technology and materials continue to develop, more effective substances for promoting axon regeneration are likely to emerge.

## 5. Conclusion

In conclusion, this study utilized bibliometric tools to provide an overview of authors, influential journals, productive countries/organizations, popular papers, and burst keywords related to autophagy and SCI. By exploring this map, readers can better understand the relationship between autophagy and SCI, promote international collaboration among relevant organizations or countries, and guide scholars in selecting cooperative partners to accelerate the process of research in autophagy and SCI. With concerted efforts, future research will uncover potential and effective targets for treatment.

## Acknowledgments

Thanks to Shanghai HaoSheng Biotechnology Co., Ltd. for providing language editing services.

## Author contributions

**Conceptualization:** Zhenyu Zhang.

**Visualization:** Yangjun Xu, Wei He, Zhongwei He, Jinxiang Shang.

**Writing – original draft:** Fei Wang, Zhenyu Zhang.

**Writing – review & editing:** Songou Zhang, Xiang Wang.
